# The relationship of the neo-angiogenic marker, endoglin, with response to neoadjuvant chemotherapy in breast cancer

**DOI:** 10.1038/sj.bjc.6603491

**Published:** 2006-12-12

**Authors:** M J Beresford, A L Harris, M Ah-See, F Daley, A R Padhani, A Makris

**Affiliations:** 1 Academic Oncology Unit, Mount Vernon Cancer Centre, Northwood, Middlesex, HA6 2RN, UK; 2 Cancer Research UK Molecular Oncology Laboratories, John Radcliffe Hospital, Oxford, UK; 3 Gray Cancer Laboratory, Rickmansworth Road, Northwood, Middlesex, UK; 4 Paul Strickland Scanner Centre, Mount Vernon Hospital, Northwood, Middlesex, UK

**Keywords:** endoglin, CD105, breast cancer, predictive markers, neoadjuvant chemotherapy

## Abstract

Endoglin (CD105) is upregulated in endothelial cells of tissues undergoing neovascularisation. A greater number of CD105-positive vessels predicts poor survival in breast cancer. We examine whether CD105 expression predicts response to neoadjuvant chemotherapy. Fifty-seven women (median age 50 years, range 29–70) received neoadjuvant chemotherapy for operable breast cancer. Immunohistochemical staining using monoclonal antibodies to CD105 and CD34 was performed on pretreatment biopsies and post-treatment surgical specimens. Individual microvessels were counted in 10 random fields at × 200 magnification. Median counts were correlated with clinical and pathological response using the Mann–Whitney *U*-test. Forty-five out of fifty-seven patients (79%) responded clinically, 22 (39%) responded pathologically. On pretreatment biopsies, clinical responders had significantly lower median CD105-positive vessel counts than nonresponders (median counts 5 and 9.3/high-power field (hpf), median difference=4.0/hpf, 95% CI 0.5–8.0/hpf, *P*=0.02). For pathological responders and nonresponders, median counts were 4.8 and 5.5/hpf (median difference –0.5/hpf, 95% CI=−2.5–2.0/hpf, *P*=0.77). CD34 expression (total microvessel density) did not correlate with response. Pretreatment CD105 expression predicts for clinical response to chemotherapy, with a lower initial count being favourable. Patients with high baseline new vessel counts or increased counts after conventional therapy may benefit from additional antiangiogenic therapy.

The formation of new blood vessels, angiogenesis, is a requirement for the growth of tumours and for the development of metastases (Folkman, 1990). Angiogenic activity is heterogeneous within a tumour type, and surrogate markers such as microvessel density are commonly used to quantify angiogenesis. Microvessel density can be scored by immunohistochemistry using antibodies against factor VIII-associated antigen, PECAM-1/CD 31 or CD 34. This technique was developed by Weidner *et al* (1991), initially using antibodies against factor VIII-related antigen that stain mainly mature vessels. The more recent use of antibodies against CD31 (platelet endothelial cell adhesion molecule) and CD34 also stain immature vessels and are thus referred to as panendothelial markers. The assessment of microvessel density has been related to the presence of metastases (Weidner *et al*, 1991) and overall survival in breast cancer (Bosari *et al*, 1992). A meta-analysis of 43 independent studies by Uzzan *et al* (2004) revealed that a high microvessel density significantly predicts for a poor survival (relative risk=1.54, 95% CI=1.29–1.84).

Endoglin (CD105) is a cell-surface glycoprotein that binds with high affinity to transforming growth factors (TGF) *β*1 and *β*3 (Cheifetz *et al*, 1992) which are involved in regulation of cell differentiation and proliferation in most cell types. The association of CD105 with angiogenesis initially came from Wang *et al* (1993) who found that monoclonal antibodies to CD105 reacted strongly with the endothelium in various tumour tissues but only weakly in normal tissues. CD105 is overexpressed on endothelial cells of tissues undergoing neovascularisation and is strongly expressed in breast cancer (Bodey *et al*, 1998). Blood vessels in several normal human tissues used as controls contained significantly lower levels of CD105 in accordance with the slow turnover of normal endothelial cells in comparison with tumour cells. Unlike CD31 and CD34, which stain both mature and immature vessels, CD105 appears to be much more specific for new, immature vessels.

There is evidence that conventional neoadjuvant chemotherapy can reduce the microvessel density in primary breast cancer (Makris *et al*, 1999; Bottini *et al*, 2002). Staining of surgical histopathological samples with CD34 monoclonal antibody in patients who had received neoadjuvant chemotherapy was compared with staining of samples from patients who had undergone primary surgery to be later followed by adjuvant chemotherapy. The median scores of microvessel staining were significantly lower in the neoadjuvant group (Makris *et al*, 1999). This raises the possibility that chemotherapy directly affects angiogenesis, although the reduction in microvessel density may be secondary to tumour regression caused by the neoadjuvant treatment. The purpose of this study was to investigate whether CD105 staining is influenced by neoadjuvant treatment of breast cancer with chemotherapy and whether pretreatment CD105 staining on biopsy specimens is predictive of response to chemotherapy. A comparison of new vessel counts (CD105 expression) and total vessel counts (CD34 expression) was also planned.

## MATERIALS AND METHODS

Fifty-seven female breast cancer patients were recruited prospectively and consecutively from the Mount Vernon cancer network. The study was open to all women about to undergo neoadjuvant chemotherapy for primary operable breast cancer for tumours larger than 2 cm in diameter at presentation (T2-4, N0-2, M0). As part of routine management, all patients underwent a trucut or mammotome biopsy to confirm the diagnosis before commencing treatment. Participants were consented to allow additional immunohistochemical examination of the tissue excised at biopsy and at definitive surgery. Twenty-two of the patients were recruited as part of a concurrent study of the role of dynamic magnetic resonance imaging in predicting response to neoadjuvant chemotherapy. Patients were scheduled to receive six cycles of 5-fluorouracil, epirubicin and cyclophosphamide chemotherapy on a 21-day cycle. Doses used were 5-fluorouracil 500 mg m^−2^, epirubicin 75 mg m^−2^, and cyclophosphamide 500 mg m^−2^.

Immunohistochemical staining for CD105 was performed retrospectively on pretreatment biopsy specimens and post-treatment surgical specimens. The formalin-fixed paraffin-embedded tumour specimens were dewaxed and rehydrated through graded alcohols. Following rinsing in Tris-buffered saline Tween, endogenous peroxidase was blocked using Dako peroxidase block and a protein block was applied. CD105 antibody (DakoCytomation, Cat No. M3527, Glostrup, Denmark) diluted to 1/1000 in Dako S2022 antibody diluent was applied and the specimen incubated for 1 h. This is a longer incubation time than recommended by the Dako kit, but we have previously found that a shorter time results in weak or negative staining. The following antibodies and reagents were each applied for 15-min incubations with Tris buffer washings in between: anti-mouse IgG-IgG-HRP, amplification reagent and anti-fluorescein-HRP. Following DAB substrate application and a further 5-min incubation, the specimens were rinsed in water, counterstained with haematoxylin, dehydrated through alcohol and mounted. Isotopic IgG controls were performed with all runs; positive controls were breast and tonsil tumours.

Microscope fields were observed at a magnification of × 200 (field of view=493.1 × 368.5 *μ*m^−1^) and the number of vessels staining positive for CD105 on a single field were counted. The next field was chosen randomly but systematically by moving the view on by five field widths. This process was repeated so that vessel counts were obtained for 10 random fields. For small biopsy specimens of less than 10 fields in size, the whole specimen was counted. The median value was calculated for each specimen and expressed as count per high-power field (hpf). One observer, with no knowledge of patient details or outcomes, performed the main analysis. Although the observer was clearly aware of which were the biopsies and which were the surgical specimens, he had no knowledge of the responder status of the patient. A second observer independently scored 10 of the specimens (chosen at random) using the same technique to assess interobserver variability. Furthermore, additional sections from 10 of the specimens were stained on a different date and counted separately to assess the reliability of the staining methods.

Clinical response was determined by physical examination in outpatient clinics. Patients were routinely examined before each cycle of chemotherapy and were judged to be clinical responders if at the end of the course of chemotherapy, the tumour had shrunk in longest diameter by 50% or more from the initial prechemotherapy measurement. All patients also underwent mammography and/or ultrasound examinations before commencing therapy and on completion of chemotherapy, but for the purpose of this study clinical response was determined by examination alone.

A surgical specimen was declared to show a pathological response if there was evidence of an apparent reduction in tumour cell-to-stroma ratio or if the following chemotherapy-induced changes were observed: (1) enlarged cells, (2) finely vacuolated, voluminous cytoplasm, (3) an enlarged vesicular nucleus with a prominent single eosinophilic nucleolus, (4) occasionally an enlarged hyperchromatic dense nucleus with an irregular outline, (5) compact hyalinized fibrous tissue at the site of good tumour response. Surgical specimens that demonstrated none of these features were declared to be non-responders pathologically.

Median CD105 counts were compared for pretreatment *vs* post-treatment specimens. Counts were then grouped into clinical responders and nonresponders and pathological responders and nonresponders. These groups were then analysed using the Mann–Whitney *U*-test for nonparametric data (StatsDirect™, Cheshire, UK) and two-sided *P*-values obtained. Absolute change of paired pretreatment biopsy and post-treatment surgery specimens was evaluated using Wilcoxon's signed-rank test for the group as a whole and by clinical and pathological response category. In addition, the percentage change in CD105-positive vessel counts from biopsy to surgery was calculated and response groups were again compared using the Mann–Whitney *U*-test.

Specimens were also stained using anti-CD34 antibody (Novocastra Labs Ltd, Newcastle, UK, NCL-L-END™) using a similar technique to that described for the CD105 antibody and total vessel counts were obtained using the same counting methods. Median baseline CD34 counts were analysed by clinical and pathological response categories and differences between the groups assessed using the Mann–Whitney *U*-test. For each specimen, an estimate of the proportion of ‘activated vessels’ (CD105 counts/CD34 counts) was expressed as a percentage, and these percentage values were again compared by response category.

## RESULTS

Table 1 shows the patient and tumour characteristics for the 57 women in the study. Forty-five of the 57 patients (79%) were judged to have had a clinical response, whereas 22 patients (39%) had a pathological response according to the aforementioned criteria. Four patients did not proceed to surgery – three had a complete clinical and radiological response and were offered the option of no surgery, whereas the fourth patient declined surgery despite having no discernable clinical response. These patients were still included in the analysis of clinical response and biopsy vessel staining, but could not be analysed for pathological response.

**Figure 1:**
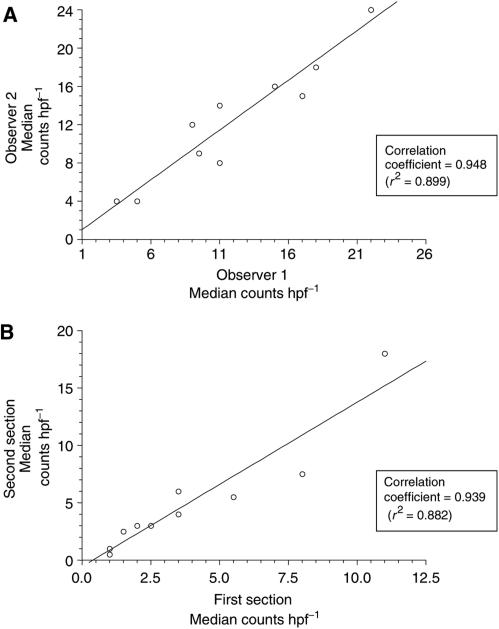
(**A**) Comparison of CD105-positive vessel counts per hpf for observer 1 and observer 2 on 10 randomly chosen specimens. (**B**) Comparison of CD105-positive vessel counts per hpf for different sections of the same specimen for 10 randomly chosen cases.

On the prechemotherapy biopsy specimens, the number of fields counted ranged from four to 10 (median=10). The median CD105-positive vessel counts per field ranged from 0 to 18.5 (median=4). For the postchemotherapy surgical specimens, 10 fields were counted in all patients, with a CD105-positive vessel count range of 0–38 (median=3). Interobserver variability was assessed on 10 specimens counted by both observers. Figure 1A shows the comparison of vessel counts by each observer, giving a correlation coefficient of 0.948 (*r*^2^=0.899). Similarly, correlation coefficients were determined for vessel counts from stains performed on alternative sections from 10 specimens (0.949, *r*^2^=0.882) and this comparison is shown in Figure 1B.

**Figure 2:**
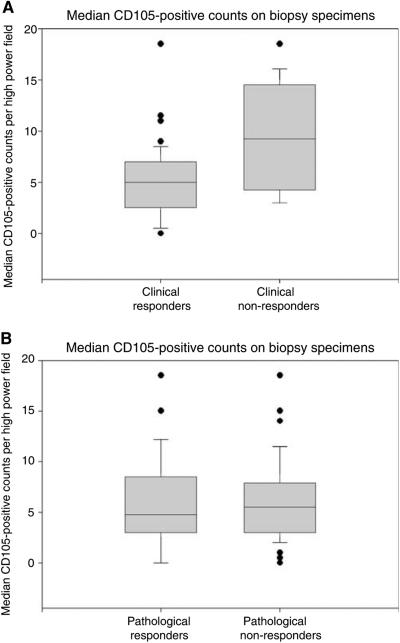
Box and whisker plots showing the distribution of median CD105-positive vessel counts per hpf from pretreatment biopsy specimens grouped by (**A**) clinical response and (**B**) pathological response. Mann–Whitney *U*-test for differences in distributions (**A**) *P*=0.02, (**B**) *P*=0.77.

Comparison of biopsy specimens from clinical responders (*n*=45) and nonresponders (*n*=12) revealed median CD105 counts of 5 and 9.3/hpf, respectively (Mann–Whitney *U*-test for probability of difference in distributions *P*=0.02, see Table 2). On pretreatment biopsies, there was no significant difference in CD105 staining between pathological responders and nonresponders (median counts of 4.8 and 5.5/hpf respectively, *P*=0.77). Figure 2 shows the distribution of CD105 counts from biopsy specimens, grouped for both clinical and pathological response.

For analysis of the postchemotherapy surgical specimens, eight patients were excluded. Four of these did not undergo surgery for reasons outlined above and a further four had a complete pathological response and hence had no tumour to stain. Fifteen of the remaining patients were classified as pathological responders and 34 as nonresponders. The median CD105-positive vessel counts on the surgical specimens are shown by response category in Table 2.

Comparison of individual paired results from biopsy and surgical specimens using Wilcoxon's signed-rank test revealed a median difference of 0.25/hpf (95% CI –1.75 to 2.25/hpf, *P*=0.81) for the whole group, excluding the four nonsurgery and four complete pathological response patients. In clinical responders, the median difference between biopsy and surgical vessel counts was −0.25/hpf (95% CI –2.25 to 1.75/hpf, *P*=0.81) and in clinical nonresponders it was 2.05/hpf (95% CI –8.75 to 8.0/hpf, *P*=0.52). In pathological responders, the median difference was 2.13/hpf (95% CI 0.0 to 4.5/hpf, *P*=0.057) and in nonresponders it was –0.75/hpf (95% CI –3.5 to 2.0/hpf, *P*=0.51).

**Figure 3:**
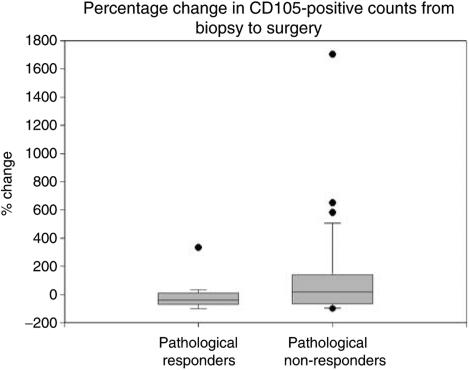
Box and whisker plot of the percentage change from prechemotherapy biopsy to post-chemotherapy surgical CD105-positive vessel counts, grouped by pathological response. Mann–Whitney *U*-test for differences in distributions *P*=0.11.

An analysis of the percentage change in CD105-positive vessels from pretreatment biopsy to post-treatment surgical specimens reveals a mean decrease of 11% in pathological responders (95% CI −69.3 to 47.1%). Pathological nonresponders had a mean *increase* in CD105 vessels of 129.1% from biopsy to surgical specimens (95% CI 8.2–250.1%). This difference did not achieve significance on the Mann–Whitney *U*-test (*P*=0.11). The four pathological complete responders necessarily had a 100% decrease in CD105-positive vessels (owing to no tumour remaining for assessment) and were excluded from this analysis. The percentage change in CD105-positive staining is represented in Figure 3.

The results for CD34 staining are shown grouped by response category alongside those for CD105 in Table 2. A ratio of new to total vessel counts (CD105-positive counts/CD34-positive counts) was obtained for each patient and expressed as a percentage. Table 3 shows the median ‘activated vessel’ percentages for biopsy and surgical specimens grouped by clinical and pathological response assessments. It can be seen that this ratio does not differ between responders and nonresponders. Paired comparisons of the ‘activated vessel’ ratio between biopsy and surgery specimens using Wilcoxon's signed-rank test revealed a median difference of 4.1% (95% CI –11.5 to 20.4, *P*=0.61).

## DISCUSSION

Endoglin (CD105) is a 180-kDa integral membrane glycoprotein that binds with high affinity to TGF *β*1 and *β*3 (Cheifetz *et al*, 1992). Transforming growth factor *β* is a member of a group of growth factors involved in inhibition and regulation of cell differentiation and proliferation in most cell types via interaction with transmembrane serine and threonine kinase receptor complexes (Massague, 1990). CD105 binds TGF*β* and modulates signalling by antagonising some of its effects. In CD105-deficient cells, the inhibitory effects of TGF*β* on proliferation and migration were found to be enhanced (Li *et al*, 2000a, 2000b), thus implying that abundant expression of CD105 might shield cells from the inhibitory effects of TGF*β*.

In breast cancer tissue, CD105 expression is inversely correlated with both overall and disease-free survival. Kumar *et al* (1999) assessed microvessel density in 106 patients using monoclonal antibodies to CD105 and the panendothelial marker CD34. CD105 expression correlated significantly with overall (*P*=0.0029) and disease-free (*P*=0.0362) survival, whereas CD34 counts showed no such relationship (Kumar *et al*, 1999). Dales *et al* (2004) examined the prognostic significance of CD105 and CD31 immunocytochemical expression in 905 breast cancers. A greater number of CD105-positive microvessels correlated significantly with a poorer survival (*P*=0.001) both on uni- and multivariate analysis. This correlation was also seen in a subgroup of node-negative patients (*P*=0.035). CD31 expression correlated with poor survival, but not in the node-negative subgroup and not on multivariate analysis (Dales *et al*, 2004). It should be noted, however, that there is cross-reactivity of CD31 antibodies with fibroblasts and plasma cells leading to a degree of over-staining, which is not seen when using the more specific CD34 (Leek, 2001). CD105 might turn out to be a superior prognostic indicator to established panendothelial markers. Given that CD105 has been known as an endothelial marker for some time now, it is surprising that it has not been more extensively investigated.

The aim of our study was to take this as a step further to see whether the poor prognosis suggested by a high CD105 count could be related to a poor response to chemotherapy treatment. Neoadjuvant chemotherapy allows useful assessment of tumour response to treatment, both clinically and pathologically, and we could therefore observe changes in CD105 staining in relation to response. We show that clinical responders are more likely to have lower baseline CD105-positive vessel counts than clinical nonresponders, and thus that high CD105 counts might be a predictor of a poor response to chemotherapy. However, assessment of pathological response did not appear to be related to pretreatment CD105 counts.

In patients with a good pathological response, the post-treatment median CD105-positive count was lower than those with no pathological response, but this did not achieve significance. It should be noted that the best (complete) pathological responders were excluded from this part of the analysis by necessity as there was no remaining tumour to stain. The median counts reported for ‘pathological responders’ might therefore be falsely high, thus resulting in a loss of significance.

Analysis of the percentage changes in positive staining between pre- and post-treatment specimens reveals an interesting trend. There is a tendency for pathological responders to show a modest decrease in CD105 staining, whereas nonresponders show an increase. The wide confidence limits prevent this from achieving significance, but again the complete responders are excluded.

We found that although there was a trend for lower pretreatment CD34 expression in clinical or pathological responders, unlike with CD105 these values did not achieve statistical significance. This gives further evidence that CD105 is a superior predictive marker to CD34/31 total microvessel density. Our analysis of the ratio of ‘activated’ or new vessels to total vessels (CD105 counts/CD34 counts) did not show any significant correlations with response, nor did the ratio change significantly after chemotherapy treatment. It should be noted that from the 18 statistical analyses performed in this study, the only one to achieve statistical significance was in relation to clinical response assessment and baseline CD105 counts. The possibility of chance findings must be considered, and it should also be noted that some of the other tests showed strong trends that failed to achieve statistical significance, but may be worth examining with a larger sample group.

Assessment of endoglin may have other applications in the management of breast cancer. For example, elevated levels of soluble CD105 have been found in the plasma of breast cancer patients when compared with normal controls, and particularly high levels have been linked with metastatic disease (Li *et al*, 2000a, 2000b). Furstenberger *et al* (2006) recently looked at changes in serum levels of soluble angiogenesis-related factors in breast cancer patients receiving anthracycline or taxane-based neoadjuvant chemotherapy. Although there were no differences in baseline levels of soluble CD105 between patients and controls, they found that patients demonstrated significant decrease in circulating levels of soluble CD105 after two cycles of chemotherapy (*P*<0.01).

With the emergence of new data on the efficacy of antiangiogenic agents in breast cancer (Thorpe, 2004), it has become more important to assess tumour vasculature and the relationship with conventional cytotoxic agents. Our findings fit with recent evidence to suggest that newly activated vessels may be structurally and functionally abnormal. Jain (2005) showed that new vessels are leaky, tortuous and have haphazard connections and irregular basement membranes. These abnormalities lead to heterogeneity in tumour blood flow and thus to interstitial hypertension, hypoxia and metabolic disturbance. This in turn might result in poor drug delivery to the tumour, thus explaining why patients with higher baseline CD105 expression might have a worse response to chemotherapy (and is consistent with the poor prognosis found to be associated with high counts in other studies).

In summary, this study suggests that a lower baseline CD105-positive vessel count is favourable for a good clinical response to conventional chemotherapy. The trend towards increased CD105 expression after neoadjuvant chemotherapy in pathological nonresponders could be explained by the induction of angiogenesis in resistant tumours. The introduction of antiangiogenic agents such as bevacizumab in this setting should be investigated in prospective studies.

## Figures and Tables

**Table 1:** Patient and tumour characteristics

Number of patients	57
	
*Age (years)*	
Median	50
Range	29–70
	
*Initial tumour stage*	
T1	0
T2	37
T3	15
T4	5
N0	30
N1	23
N2	2
N3	2
	
*Endocrine status*	
ER +ve/PR +ve	29
ER −ve/PR −ve	16
ER +ve/PR −ve	5
ER −ve/PR +ve	1
ER +ve/PR unknown	4
Unknown	2
	
*Grade*	
1	1
2	23
3	27
Unknown	6

**Table 2:** Median CD105 and CD34-positive vessel counts per hpf for biopsy and surgical specimens grouped by clinical and pathological response categories

	**Response**	**CD105 counts/hpf**			**CD34 counts/hpf**		
	**assessment**	**Responders**	**Nonresponders**	**Median difference^a^**	**Responders**	**Nonresponders**	**Median difference^a^**
Biopsy specimens	Clinical	5.0	9.3	−4.0 (−8.0 to −0.5)	10.5	17.0	−5.0 (−9.5 to1.0)
				*P*=0.02			*P*=0.09
	Pathological	4.8	5.5	−0.5 (−2.5 to 2.0)	3.0	5.8	−2.5 (−5.5 to 0.5)
				*P*=0.77			*P*=0.12
							
Surgical specimens	Clinical	5.0	4.0	−0.5 (−6.5 to 3.0)	12.5	12.0	−0.5 (−8.0 to 5.0)
				*P*=0.40			*P*=0.93
	Pathological	3.0	5.8	−2.5 (−5.5 to 0.5)	13.5	11.3	1.5 (−4.0 to 6.0)
				*P*=0.12			*P*=0.60

a Median differences between responders and nonresponders are shown with 95% CI in parentheses and Mann–Whitney *U*-test *P*-values for differences in distributions.

**Table 3:** Percentage of activated vessels (CD105/CD34 ratio) on biopsy and surgical specimens grouped by clinical and pathological response assessments

		**% Activated vessels (CD105/CD34 ratio)**		
	**Response assessment**	**Responder (%)**	**Nonresponder (%)**	**Median difference^a^**
Biopsy	Clinical	40.7	47.7	−3% (−25.2 to 25.0)
				*P*=0.65
	Pathological	32.0	46.4	0% (−21.0 to 18.5)
				*P*=0.94
				
Surgery	Clinical	34.8	32.2	0% (−22.2 to 24.5)
				*P*=0.93
	Pathological	26.9	38.9	−8.9% (−28.6 to 10.7)
				*P*=0.36

a Median differences between responders and non-responders are shown with 95% CI in parentheses and Mann–Whitney *U-*test *P-*values for differences in distributions.
